# Canine seroprevalence to *Orientia* species in southern Chile: A cross-sectional survey on the Chiloé Island

**DOI:** 10.1371/journal.pone.0200362

**Published:** 2018-07-06

**Authors:** Thomas Weitzel, Ju Jiang, Gerardo Acosta-Jamett, Constanza Martínez-Valdebenito, Javier López, Allen L. Richards, Katia Abarca

**Affiliations:** 1 Laboratorio Clínico, Clínica Alemana de Santiago, Facultad de Medicina Clínica Alemana, Universidad del Desarrollo, Santiago, Chile; 2 Viral and Rickettsial Diseases Department, Naval Medical Research Center, Silver Spring, MD, United States of America; 3 Instituto de Medicina Preventiva Veterinaria y Programa de Investigación Aplicada en Fauna Silvestre, Facultad de Ciencias Veterinarias, Universidad Austral, Valdivia, Chile; 4 Departamento de Enfermedades Infecciosas e Inmunología Pediátricas, Escuela de Medicina, Pontificia Universidad Católica de Chile, Santiago, Chile; 5 Hospital Veterinario Puente Alto, Santiago, Chile; 6 Millennium Institute on Immunology and Immunotherapy, Escuela de Medicina, Pontificia Universidad Católica de Chile, Santiago, Chile; Johns Hopkins University, UNITED STATES

## Abstract

**Background:**

Scrub typhus is a potentially life-threatening vector-borne infection caused by *Orientia* species. It occurs mainly in the Asian-Pacific region, where it causes significant morbidity and mortality. Recently, an endemic focus of scrub typhus has been described in South America, on Chiloé Island in southern Chile. Dogs have been used as sentinel hosts to determine the presence and spatial distribution of various vector-borne infections. Their suitability to gain insight into human exposure to *Orientia tsutsugamushi* has been suggested in studies from Asia.

**Methodology:**

In January 2016, we conducted a cross-sectional study, which included the two main cities on Chiloé Island. Canine blood samples were obtained in households, chosen by double stratified random sampling in urban and by convenience in rural locations. Specimens were tested by ELISA for IgG antibodies against whole-cell antigen preparations from three strains of *O*. *tsutsugamushi*. Data were further analyzed for factors associated with seropositivity including spatial clustering.

**Results:**

Serum samples from 202 dogs (104 urban, 98 rural) were tested for IgG against *O*. *tsutsugamushi*, of which 43 (21.3%) were positive. Seroprevalence rates were higher in rural than in urban settings (*p*<0.01) and in older compared to younger dogs (*p*<0.01). Spatial analysis by LISA indicated the presence of four localities of highly grouped cases.

**Conclusions:**

The detected seroprevalence supports the endemicity of scrub typhus in southern Chile and suggests a wide exposure of household dogs to the infected, yet unknown vector(s). The spatial data will be used for future research identifying further human cases as well as the local vector(s)/reservoirs for scrub typhus in southern Chile. The study reinforces that dogs are useful sentinels for *Orientia* spp. in regions of uncertain endemicity and distribution.

## Introduction

Scrub typhus is a vector-borne zoonosis caused by *Orientia* species that typically manifests as a febrile disease with or without eschar and/or rash and has a potentially severe outcome [1]. Although widely under-recognized and under-diagnosed, it is considered the most important rickettsial infection worldwide [2]. Until recently, scrub typhus was associated with a single species, *Orientia tsutsugamushi*, which exclusively occurred within the so-called ‘tsutsugamushi triangle’ ranging from Pakistan in the West, far-eastern Russia in the East to northern Australia in the South [1]. But now with two single cases of the disease observed in 2006, this epidemiological paradigm has been reevaluated. One of the patients was infected in the Middle East by a new *Orientia* pathogen, named *Candidatus* Orientia chuto [3], the second case was observed in a Chilean traveler returning from the Chiloé Archipelago in southern Chile [4]. In 2015 and 2016, our group was able to prove further autochthonous scrub typhus cases in the same region [5]. Until now, many aspects of this new infectious disease in South America including the spectrum of causative *Orientia* species/strains as well as the vectors and zoonotic reservoirs are unknown; still, this finding has important global implications suggesting a much wider geographic distribution of scrub typhus than previously known [6,7].

Since dogs share the same environment and are co-exposed to the same arthropod vectors as their human owners, they are useful sentinel hosts for human diseases [8,9]. This “One Health” principle has been applied in various seroepidemiological surveys to analyze spatial and temporal aspects of tick-borne and other zoonotic pathogens. Dogs are also susceptible to *O*. *tsutsugamushi* infection and their suitability to gain insight into human exposure has been suggested in studies from endemic areas in Asia [10–12]. Our study aimed to analyze the prevalence, spatial distribution, and associated factors of seropositivity to *Orientia* antigens in household dogs from the Chiloé Island in southern Chile.

## Methods

A cross-sectional study was conducted in January 2016 in urban and rural areas of Ancud and Castro, the two main cities of the Chiloé Island. Households were chosen by double stratified random sampling per building block in urban and by convenience in rural locations, as described previously [13]. After owners signed informed consent, one dog per household was examined by a veterinarian and blood samples were obtained. Demographic and health information on dogs and their owners were collected by a standardized questionnaire [14] and household locations were recorded using a GPS device. Serum samples were separated from clotted blood and kept at -20°C until shipment to the Naval Medical Research Center (Silver Spring, MD, USA), where they were processed in a blinded manner. Specimens were assessed at 1:100, 1:400, 1:1600, and 1:6400 dilutions for IgG against a mixture of whole-cell antigen preparations from *O*. *tsutsugamushi* Karp, Kato and Gilliam strains in an ELISA as described previously [15], except that goat-anti-dog IgG HRP (KPL, Gaithersburg, MD, USA) was used as secondary antibody; this assay has been shown previously to be specific for *Orientia* species [3, 4, 16]. Samples with a total net absorbance ≥1.000 were considered positive with the titer defined as the inverse of the highest dilution with an OD of ≥0.2 [17]. Serum samples from dogs (n = 5; 3 negative and 2 spotted fever group rickettsia positive samples with antibody titers of 1600 and 6400) of a non-endemic region (USA) were tested to assure the level of non-specific seroreactivity for dog sera. All 5 samples were negative (total net absorbance <1.000) by the *Orientia*-specific ELISA (data not shown).

To assess factors associated to seropositivity, unconditional logistic regressions analyses of variables of household (education, number of persons, number of dogs, husbandry practices), location (city, setting), and dog (sex, breed, age, anti-parasitic treatment, presence of ticks or fleas) were carried out, followed by a multivariable GLM model with binomial errors. Factors with a likelihood-ratio test *p*-value <0.15 were used for a multivariable logistic regression. The fit of the fixed-effect model was assessed using the area under the curve (AUC) of the receiver-operating characteristic (ROC) and Hosmer-Lemeshow goodness-of-fit test [18]. Regression analysis to identify influential covariate patterns was carried out by plotting the Pearson’s residual squared (Δχ^2^), the influence (Δβ), and delta D (ΔD) against the predicted probabilities of being seropositive as suggested by Hosmer and Lemeshow [18]. The diagnostic parameters ΔD and Δχ^2^ determine the effect of each covariate pattern on the fit of the model by measuring the change in the deviance or χ^2^ residual, while Δβ measures the effect of each covariate pattern on the value of the estimated parameters. All statistical analyses were carried out in R version 3.4.1 [19]. Additionally, we assessed clustering of seropositive dogs using Nearest Neighbor test, Moran test, and Local indicators of spatial association (LISA test) in ArcGis 10.1. Finally, clustering was further investigated by Cuzick and Edwards' test for inhomogeneous populations. In this analysis, binary data (seropositive, negative) and up to the 6^th^ nearest neighbor were considered. The significance of spatial clustering was assessed by calculating a z-statistic [20].

### Ethics statement

The study was approved by the Ethics Committee on Animal Welfare in Research, Faculty of Medicine, Pontificia Universidad Católica de Chile (Protocol #12–033), in accordance with the Terrestrial Animal Health Code of the World Organisation for Animal Health (OIE, 24^th^ Edition, 2015), the Directive 2010/63/EU on the protection of animals used for scientific purposes, and the Chilean Law 20.380 on Animal Protection (2009).

## Results

A total of 202 dogs were included, 104 from urban and 98 from rural areas. Most dogs were infested with fleas, whereas ticks and mites were detected in five and two dogs, respectively. A total of 43 dogs (21.3%) were seropositive for *Orientia tsutsugamushi* with titers from 400 to 1600. Seroprevalence rates were similar in the two cities studied, but higher in rural than in urban areas (Table 1). Univariable logistic regression analysis, which included 12 variables, demonstrated that “Rural Setting” (vs. “Urban Setting”) and “Age ≥ 24 months” (vs. “Age <24 months”) were associated with seropositivity (Table 2). Final analysis by the multivariate model using these two categorical variables confirmed that dogs from rural areas and those older than 24 months were 3.1- and 3.4-times more likely to be seropositive, respectively (Table 3). The Hosmer-Lemeshow test indicated adequate regression model fit (p>0.05).

**Table 1:** Canine seropositivity rates to *Orientia*–specific antigens in study cites on Chiloé Island and in previous studies from Asia.

Study region	N	Positive	Prevalence	95% CI
Chiloé				
Ancud	100	22	22.0%	15.0–31.1
Rural	48	13	27.1%	16.6–41.0
Urban	52	9	17.3%	9.4–29.7
Castro	102	21	20.6%	13.9–29.4
Rural	50	15	30.0%	19.1–43.8
Urban	52	6	11.5%	5.4–23.0
Rural (all)	98	28	28.6%	20.6–38.2
Urban (all)	104	15	14.4%	8.9–22.4
Total	202	43	21.3%	16.2–27.4
Vietnam [10]				
Total	64	29	45.3%	33.7–57.4
Malaysia [11]				
Rural	97	31	32.0%	23.5–41.8
Urban	97	0	0%	0–3.8
Total	194	31	16.0%	11.5–22.8
Sri Lanka [12]				
Total	123	29	23.6%	16.6–32.3

95% CI, 95% confidence interval without continuity correction

**Table 2:** Univariable Generalized Lineal Model with binomial error indicating the factors associated with *Orientia* seropositivity in dogs (n = 202) on Chiloé Island.

Factor	Positives	Negatives	OR	CI95%	p
City					
Ancud	22	78	1.00		
Castro	21	81	0.92	0.47–1.81	0.81
Setting					
Urban	15	89	1.00		
Rural	28	70	2.37	1.19–4.88	0.02*
Owner’s education					
Primary	15	38	1.00		
>Primary	28	117	0.61	0.29–1.27	0.18
Sex					
Female	14	67	1.00		
Male	29	91	1.53	0.76–3.18	0.25
Age					
<24 months	9	64	1.00		
≥ 24 months	34	94	2.57	1.19–6.04	0.02*
Pure breed					
No	31	116	1.00		
Yes	12	42	1.07	0.49–2.23	0.86
Free-roaming					
No	6	22	1.00		
Yes	37	136	0.99	0.40–2.87	0.99
Antiparasitic treatment					
No	26	105	1.00		
Yes	15	53	1.14	0.55–2.32	0.72
Presence of ticks					
No	41	155	1.00		
Yes	2	3	2.52	0.32–15.8	0.32
Presence of fleas					
No	10	32	1.00		
Yes	33	126	0.84	0.38–1.96	0.67
No of person per household			1.06	0.84–1.33	0.61
No of dogs per household			1.20	0.87–1.63	0.25

*Variables used for multivariable analysis

**Table 3:** Multivariable Generalized Lineal Model with binomial error indicating the factors associated with *Orientia* seropositivity in dogs (n = 202) on Chiloé Island.

Risk Factor	OR	95% CI	P
Site			
Urban	1.00		
Rural	3.10	1.51–6.61	<0.01
Age			
<24 months	1.00		
>24 months	3.38	1.52–8.23	<0.01

OR, odd ratio

Hosmer-Lemeshow test: AUC = 0.68; χ^2^ = 5.34, p = 0.07

**Fig 1:**
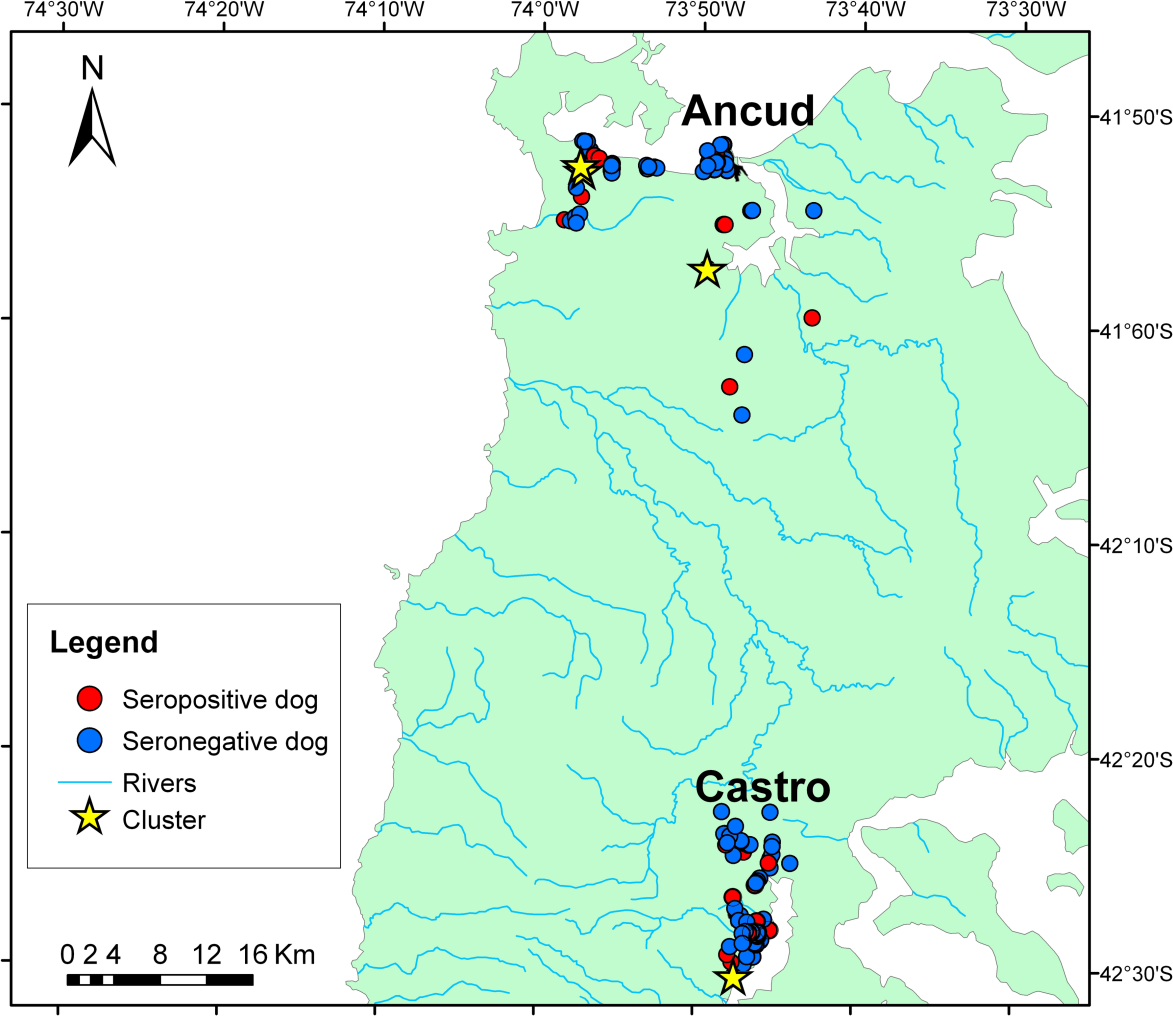
Geographical distribution of seropositive and -negative dogs and location of grouped cases by LISA analysis on Chiloé Island (Open source maps from Albers C. (2012): Coberturas SIG para la enseñanza de la Geografía en Chile. Universidad de La Frontera. Temuco. Available at: www.rulamahue.cl/mapoteca.

Spatial analysis of positive cases was negative for global clustering (Moran’s Index = –0.24, p = 0.24), but positive for local clusters (Nearest Neighbor testing, z = –6.96, p < 0.0001). The latter test, however, showed also clusters of negative cases (z = –17.5, p < 0.0001), indicating an inhomogeneous dog population in the area. Further analysis by Cuzick and Edwards’ test did not detect clustering (p > 0.05). Calculation of “Local indicators of spatial association” (LISA), however, indicated four highly grouped cases, three in rural areas of Ancud and one in rural Castro (Fig 1).

## Discussion

Sentinel surveillance of animals is an established method to detect and/or monitor human environmental and biological threats [21]. Among such sentinel animals, domestic dogs have various advantages. They are almost ubiquitous, live in close vicinity to people, are identifiable by name and owner, easily accessible and safe to sample, and can repeatedly be located for follow-up studies [21]. Canine seroprevalence studies were applied to detect zoonotic pathogens such as *Yersinia pestis* and *Francisella tularensis* [22], *Trypanosoma cruzi* [23], and various tick-borne diseases such as Lyme borreliosis [24]. Similar studies also served to monitor the spatio-temporal epidemiology of rickettsioses in endemic areas [25,26] as well as in regions of uncertain epidemiology, e.g. Germany [27], Sri Lanka [12], Brazil [28] or Australia [29]. Regarding the epidemiology of scrub typhus, dogs have only sporadically been studied, although early Japanese researchers reported them as hosts of “Akamushi” (chigger mites) and susceptible to *O*. *tsutsugamushi* infection [30–32]. Dogs have experimentally been infected with *O*. *tsutsugamushi* and strain- and dose-dependent clinical signs have been observed in the 1970s [32]. A recent report from Japan detected for the first time *O*. *tsutsugamushi* DNA in blood samples of a sick and several asymptomatic dogs [33], data which await further confirmation. Trombiculiasis (infestation with chigger mites) is a known ectoparasitosis in dogs, but the clinical relevance and range of species is uncertain since taxonomical identification of mites is challenging [34]. The concept of using dogs as sentinel hosts for scrub typhus has been applied in three surveys in Asia (Table 1). The first showed that military scout dogs in Vietnam were frequently exposed to *O*. *tsutsugamushi*, and that after 6 months of service, their seroprevalence reached >50%. Interestingly, this exposure occurred despite the regular treatment with insecticides, effectively controlling flea infestation and *Rickettsia typhi* infection [10]. Dogs from rural areas in Malaysia were seroreactive to *O*. *tsutsugamushi* in 32% of cases, with geographical variations ranging from 0% to 81%, while dogs from urban study sites were all negative [11]. The third work from Sri Lanka revealed an overall canine seroprevalence of 23.6% [12]; regions of high seropositivity were in accordance with high risk areas for human scrub typhus reported elsewhere [35]. The authors of all three studies proposed that dogs were suitable indicators for the presence of scrub typhus and useful for the surveillance of human exposure.

Dogs might also directly influence human exposure to chigger mites. A survey in Malaysia demonstrated that close contact with dogs and other pets was associated with higher rates of *O*. *tsutsugamushi* exposure. The authors proposed that dogs can serve as transport hosts for infected chigger mites, thus increasing the scrub typhus risk for their owners [36]. In our study, mite infestation was reported in two dogs. This number has to be interpreted with caution since, in contrast to larger ectoparasites such as ticks and fleas, a reliable detection and sampling of the fragile chigger mites in larger animals is difficult (without anesthesia). Furthermore, the veterinarians performing the examination in our study were not specifically trained to detect and identify parasitic mites. Vector studies of scrub typhus generally focus on smaller vertebrates such as rodents, partly because they are abundant and can be trapped, euthanized, and examined. This methodological preference, however, might bias our understanding of the complete host spectrum of trombiculid mites [37].

Although Chiloé Island has been identified as a focus of autochthonous scrub typhus [5], our understanding of this new infection in South America is only sketchy. We observed a high seroprevalence against *O*. *tsutsugamushi* antigens in dog populations of two study sites in Chiloé suggesting that the infection is endemic in the northern (Ancud) and central part (Castro) of the island. This is in accordance with the diagnosed scrub typhus cases diagnosed by our group during the last three years ([5] and unpublished data). The association of our results to the observed clinical infections suggests that seroprevalence studies in dogs are a useful surveillance tool for *Orientia* spp. in other regions of uncertain endemicity, e.g. in Chile or other countries in South America. Interestingly, the seroprevalence rates in the studies dog populations were within the same range as those reported in Sri Lanka and Malaysia [11,12]. In accordance with the latter study [11], we detected a higher exposure in dogs from rural areas. This matches the clinical experience in Asia, where the disease is also named “rural typhus” [38] and the cases in Chiloé, who to date were exclusively acquired in rural sites ([5] and unpublished data). The results of our spatial analysis of positive cases were used to determine geographical sites for ongoing studies such as rodent trapping to identify the yet unknown vectors and zoonotic reservoirs in Chile. Interestingly, one of the clusters (south of Ancud) was in very close vicinity to one of the reported human cases [5]. The recognition of hot spots is of particular relevance, since the *O*. *tsutsugamushi* infected mites commonly occurs in well-defined foci of “mite islands” [1]. Hence, our work reinforces that dog surveillance is useful to screen for the existence and spatial distribution of *Orientia* spp. in regions of uncertain epidemiology. Compared to rodents, which are usually used in non-human seroepidemiological surveys, dogs have the advantage, that they are also more convenient to sample than rodents, especially in regions with a risk of Hantavirus Cardiopulmonary Syndrome, and that they are closer related to human environments.

In conclusion, our seroepidemiological study proved the wide and cumulative exposure of household dogs to *Orientia* spp. in southern Chile, which will contribute to our knowledge of this newly discovered pathogen and its clinical and public health relevance in South America.
